# Development and validation of a heated drying air diffusion system to optimize rotary dryers and final coffee quality

**DOI:** 10.1371/journal.pone.0251312

**Published:** 2021-06-22

**Authors:** Paulo Carteri Coradi, Samuel Martens, Henrique Eguilhor Rodrigues, Andressa Fernandes Leal, Douglas Romeu da Costa, Reni Saath, Flávio Meira Borém

**Affiliations:** 1 Campus Cachoeira do Sul, Federal University of Santa Maria, Cachoeira do Sul, RS, Brazil; 2 Department of Agricultural Engineering, Federal University of Santa Maria, Santa Maria, RS, Brazil; 3 Department of Agricultural Engineering, Federal University of Sergipe, São Cristóvão, SE, Brazil; 4 Department of Agronomy, Sate University of Maringá, Maringá, PR, Brazil; 5 Department of Agricultural Engineering, Federal University of Lavras, Lavras, MG, Brazil; Tongji University, CHINA

## Abstract

The final quality of pre-processed coffees is influenced by the applied drying technology. Thus, the aim of the study was to develop and validate a heated air flow diffusion system to optimize and reduce the drying time of rotary dryers and improve the final quality of coffee. Computational fluid dynamics was used for the simulation of the air fluid dynamics in the combustion chamber of the heat generator. It was observed that the energy losses in the upper and lower walls of the heat generator chamber were higher with an increase in the convective heat transfer coefficient. It was found that the rate of fluid flow presented a fully developed profile, in which the higher speed value was found in the central region of the outlet. The reduction in moisture content during coffee drying was directly proportional to the increase in temperature. The Midilli model shows the best fit to describe the drying curves of the coffee. The effective diffusion coefficient increases with increasing temperature of the drying air. It was observed that the adjustments of the fluid dynamics in the burning of gas and the adaptation of the diffuser system significantly influenced the drying time and final quality of naturally processed and pulped coffees. In conclusion, the adapted technological set, a rotary dryer with gas heating and diffusion of heated air, had a high performance in the final quality of the coffee, and for this reason it is recommended to producers and the industry.

## 1. Introduction

The coffee culture must always seek a better interaction between productivity, quality, and reduction of production costs [1–4]. Among other factors, drying is one of the most important stages of coffee processing regarding both energy consumption and the influence that this operation has on the quality of the end product [5–7].

The drying of agricultural products can be described by mathematical models [1–3] that consider the external conditions under which the operation occurs, as well as the internal mechanisms of energy transfer and mass and their effects. In the drying, the air is heated to high temperatures and is convectively subjected to contact with the biological product causing heating of the coffee mass and the transfer of water from the inside of the product to the periphery by the principle of diffusivity, influenced by the pressure difference of steam between the drying air and the product [4–7].

The drying can cause damage to the cellular structure of the product. A reduction in the moisture content of the grains diminishes their biological activity (fungi and bacteria), physical (conductivity electrical and germination), physico-chemical changes (acid index, sugars, soluble solids, proteins, amino acids), sensory (beverage quality), and during storage possible contaminations (mycotoxins) [2–5]. Thus, the air temperature and product flow must be monitored during drying, as the variation of these parameters will interfere with the drying time and how the water diffusivity and vaporization of the coffee can change the physical and chemical characteristics of the product, reducing their quality [5–7].

The implementation of an internal program of energy conservation is the first step for a rational use of energy in a processing unit of agricultural products [8, 9]. Drying requires over 60% of the total energy used in production, whereas cultural practices consume 16%, planting and crops 12%, harvest 6%, and transport 6% [10, 11].

The rotary dryers with a radial distribution of the airflow that are available in the market were designed for the drying of coffee. Some authors have confirmed that the use of a horizontal rotary dryer with a radial distribution of airflow for drying coffee has a specific energy consumption and consumption of electrical energy approximately 90% lower than those of the regular commercial dryer. Studies on the drying of peeled coffee cherries in a rotary dryer obtained an average consumption of liquefied petroleum gas of 7.6 to 10.25 kg of gas per hour for an average time of drying of 33 and 30 h, respectively [12, 13].

Although the results obtained with a rotary dryer for drying coffee have shown some advantages, there are still numerous problems to be solved before this drying system can become viable for the producer and the industry, mainly in terms of drying capacity, system efficiency, and final coffee quality [14–17]. Therefore, an evaluation of the heating system and some adaptations in the drying set can improve the operation considerably.

This involves first the gas heater, which must guarantee the spraying and mixing of the fuel with the air [18–20]. The design of a gas heat generator depends on several factors, the main ones being fuel consumption, available fuel and air pressures in the inlet of the heat generator, temperatures, and flame length [21–23]. To decrease the flame length, it is necessary to increase the turbulence of the mixing flow, preheat the air and the fuel, increase the temperature in the chamber, decrease the fuel/air ratio, and use fuel with higher calorific value [24–26].

The commercial gas heat generators used in the drying of agricultural products have a high fuel consumption. The main problem regarding this equipment lies in adapting the burning mechanisms and the radial air flow in the drying chamber of the rotary dryer considering that, in many cases, the drying/heating generation system does not present a satisfactory performance. Therefore, the aim of this study was to develop and validate a heated air diffusion system to optimize and reduce the drying time of the rotary dryers and improve the final quality of the coffee.

## 2. Materials and methods

### 2.1 Coffee processing

The coffee was harvested manually and selectively removing only the cherry fruit from the plant. For each repetition, 800 L of the coffee variety Topazio were collected. All the raw materials were standardized by washing, separation, and manual selection of green coffees, green cane passes. The coffees were processed as natural coffee or coffee cherries, which corresponds to the coffee in fruit format, with all the parts and constitutions of the fruit, with the two cotyledones or seeds involved by the parchment or mesocarp, the pulp and the outer layer, known like bark or exocarp. The other processing corresponded to coffee without pulp, which was peeled to remove the exocarp and separate the cotyledons, and subsequently fermented to remove the mucilage or pulp. Then, natural and pulped coffees were placed on a terrace for pre-drying for two days.

### 2.2 Coffee drying

Approximately 150 L of coffee cherries were pulped and taken directly to the yard. The natural and pulped coffees were divided into distinct segments in the yard, forming a thick layer of 10 cm each, where they remained for two days. Natural coffee started pre-drying with initial moisture contents of 57% (w.b.) and after two days, it reduced to 50% (w.b.), whereas pulped coffee started pre-drying with 50% (w.b.) and after two days reduced the moisture content to 40% (w.b.), and then the beans were taken for mechanical drying. During pre-drying, the coffees were turned on the yard every two hours, remaining with a temperature in the mass between 20 to 25°C. The relative humidity of the ambient air ranged from 55 to 65% and the temperature from 20 to 23°C.

**Fig 1:**
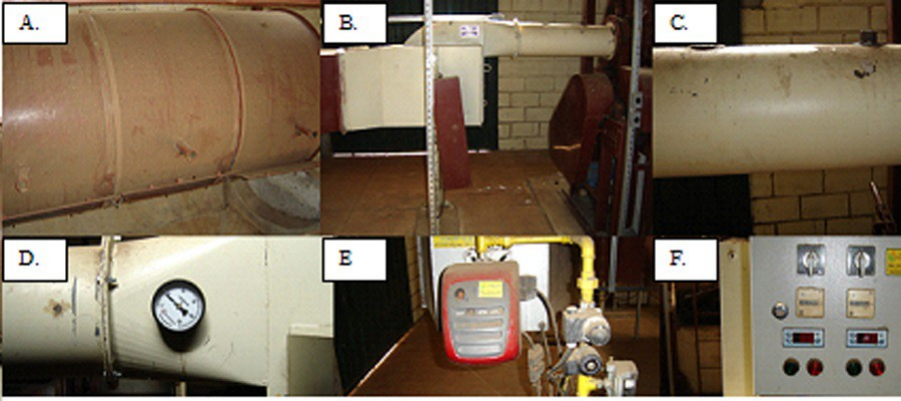
Horizontal rotary dryer (A), centrifugal fan (B), measurement of the fan outlet air temperature and dryer inlet (C), manometer to measure the air pressure exerted by the fan (D), inlet energy system (gas) and burner (E), control panel (F).

**Fig 2:**
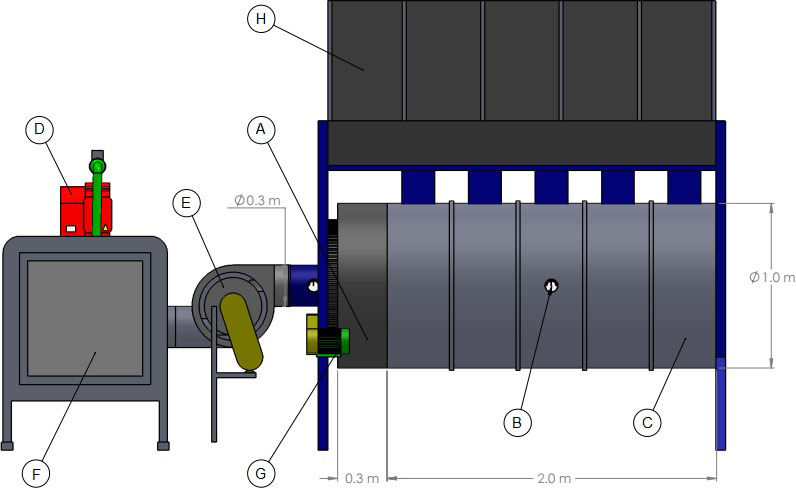
Heated air distribution and movement diffuser (A), air flow and temperature meter (B), cylindrical and rotary drying chamber (C), gas burner (D), air fan (E), flame generator and air heating (F), electric motor to move the diffuser and drying chamber (G), dryer loading (H).

A horizontal rotary dryer was used (type BE-050) (Fig 1A–1F), composed of a chamber 2.0 m of length, 1.0 in diameter, and with 5000 L capacity. It was built with 14-inch, metal galvanized, and circular. The drying air was heated using a gas burner, coupled with a centrifugal fan-diffusor with 80 m³ min^-1^ of airflow driven by a 2 HP motor, tension of 220/380 V, rotation of 1750 rpm, single phase, carbon steel structure, aluminum alloy rotor (Fig 2A–2H).

**Fig 3:**
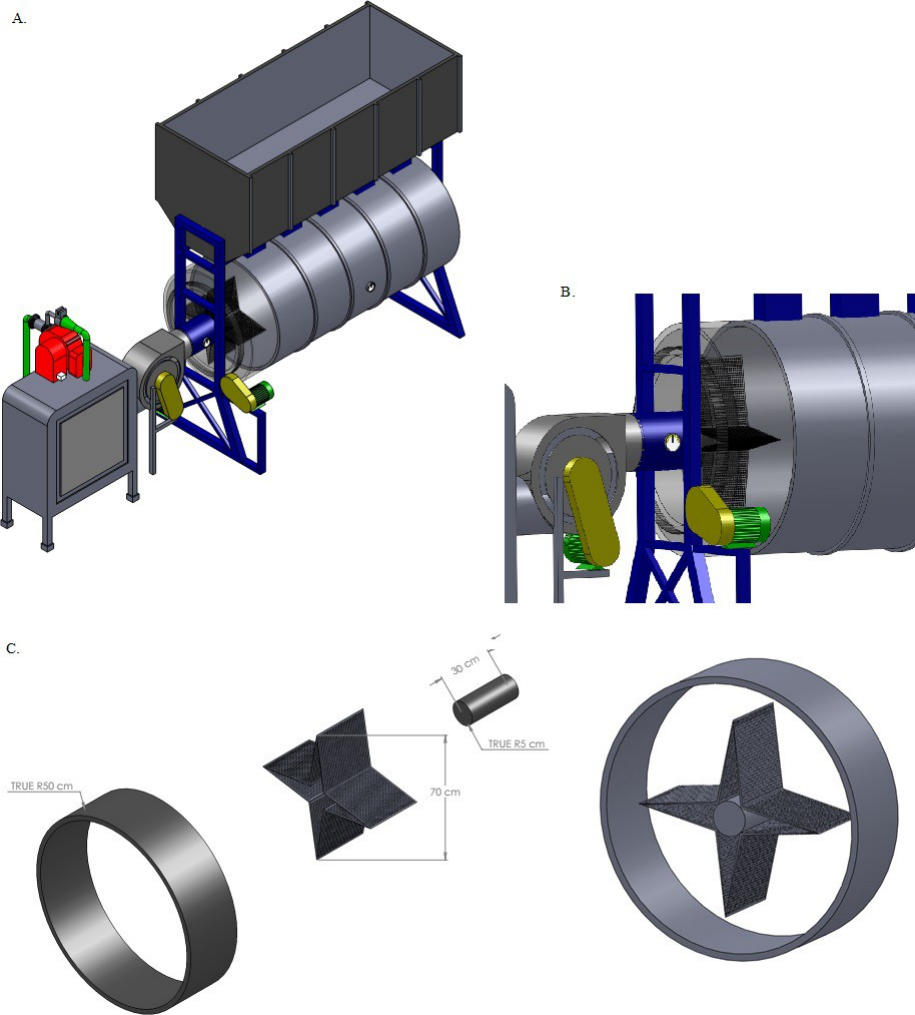
Top view of the horizontal rotary dryer, with installed diffusion system (A), development of drying air diffuser in rotary dryer (B), detail of the diffusor (C).

At the entrance of the rotary dryer, a diffusion system was adapted to direct the air flow to the bottom of the drying chamber, where the mass of coffee beans remained thicker during drying. For this purpose, the holes in the diffuser plates, alternating, were fixed consecutively (Fig 3A–3C). A smooth metal plate was placed externally, covering half of the cylindrical perimeter of the dryer, blocking the holes. This sought to ensure uniformity in the distribution of drying air throughout the process.

The drying and heating systems were controlled via sensors. To control the drying system, we used a programmable controller with the following characteristics: 5A–1x/2x 85-132/170-550 VCA, inlet—24 VCC, outlet—CPU 224 XP DC/DC/DC: 24 VCC, 14 ED 24 VCC, 10 SD 24 VCC, 4EA, 2SA, 2 common ports, micromaster 420 5 CV 17,5 A 220 V, dimensions 245 x 185 x 195 mm (H x W x D), basic operation panel (BOP). A frequency inverter type CFW 08 Vector Inverter Plus with a 220 V three-phase supply, 60 Hz frequency, with two analog inputs from 0 to 10 V and 0 to 20 mA, and 4 isolated digital inputs was used to regulate the fan airflow. The system was activated by a relay. The air flow was measured by a probe with a 16 mm reel, hot wire type, with TC type K, pluggable in a telescope, with speed air variation of 0.4 to 60 m s^-1^ and temperature of 30°C to 40°C, signals outputs from 4 to 20 mA, and operating temperature of 0 to 60°C.

The gas burner used was type AZ9, with 220 V power and 60 Hz single phase, minimum power of 321 W and maximum of 793 W, minimum pressure of 20 mBar, 8 KV and 20 mA ac, with minimum consumption of gas of 2.4 kg h^-1^ and maximum of 5.93 kg h^-1^. The gas burner was controlled by a servomotor, SWA type with electromagnetic brake, 200 RPM, rotor torque of 6.1 Nm, 1100 Watts of nominal power, electrical current of 5.2 A, mass of 7.5 kg, and 310 mm in length. A sensor SR EN 100 type was used to measure the temperature of the grain mass inside the dryer, with the characteristics of working temperature from −20°C to 150°C, a bulb type J (3 wires), a 316 stainless steel rod, and a 6 mm in diameter nylon head with fiberglass, internal ceramics isolation, and brass type material. For monitoring the temperature and relative air humidity, a QMH101 sensor was used with an HMP45D humidity and temperature probe.

The drying air temperature was measured at three points inside the rotary dryer chamber: close to the coffee layer (center), at the side end of the equipment’s drying chamber and at an intermediate point. The temperature information was sent to an external controller that passed the information to the burner to control the burning flame and to the frequency inverter to reduce or increase the air speed inside the drying chamber. The optimization and homogenization of the heated drying air was carried out by the distribution provided by the diffuser, installed after the gas fan-burner system.

**Fig 4:**
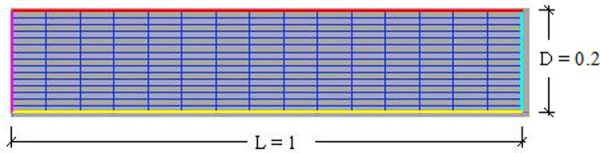
Symmetric and computational geometric mesh of the gas heat generator.

Computational fluids dynamics was used for the simulation of the combustion chamber of the heat generator. As constructive characteristics were considered the air distribution system, the controlled environmental variables, the initial and contour conditions, the dimensionless system, and the physical parameters of the air [27]. Fig 4 shows the computational geometric mesh and the symmetric of the problem in the gas heat generator.

The answer to the outlet problem and conservation of energy in the heat generator lies in the solutions of Navier–Stokes equations [27]. In this work, some considerations for the development of the mathematical model were made, such as (a) a turbulent, permanent, and two-dimensional outlet; b) the thermophysical properties of the working fluid were assumed constant. Eqs (1), (2) and (3) characterize the outlet:

Continuity equation:

$$\frac{\partial u}{\partial x}+\frac{\partial v}{\partial y}=0$$ (1)

Value momentum equation in the x-direction:

$$\frac{\partial u}{\partial t}+u\frac{\partial u}{\partial x}+v\frac{\partial u}{\partial y}=\frac{-1}{\rho }\frac{\partial p}{\partial x}+v\left(\frac{\partial^{2}u}{\partial x^{2}}+\frac{\partial^{2}u}{\partial y^{2}}\right)+\beta \left(T-T^{r}\right)\vec{g}\cdot \vec{i}$$ (2)

Value momentum equation in the y-direction:

$$\frac{\partial v}{\partial t}+u\frac{\partial v}{\partial x}+v\frac{\partial v}{\partial y}=\frac{-1}{\rho }\frac{\partial p}{\partial y}+v\left(\frac{\partial^{2}v}{\partial x^{2}}+\frac{\partial^{2}v}{\partial y^{2}}\right)+\beta \left(T-T^{r}\right)\vec{g}\cdot \vec{j}$$ (3)

where

*u*: speed in (*x)* direction (m s^-1^)
*v*: speed in (*y)* direction, in m s^-1^
*x*: coordinate in the horizontal direction (m)
*y*: coordinate in the vertical direction (m)
*p*: density (kg m^-^³)
δ: gradient;
β: volumetric expansion coefficient
T: temperature (°C)
i: unit vector in (x)
j direction: unit vector in (y) direction
Energy conservation Eq (4):

$$\frac{\partial T}{\partial t}+u\frac{\partial T}{\partial x}+v\frac{\partial T}{\partial y}=\alpha \left(\frac{\partial^{2T}}{\partial x^{2}}+\frac{\partial^{2T}}{\partial y^{2}}\right)$$ (4)

where

*α*: thermal diffusivity (m² s ^-1^)

In the energy conservation equations above, the physical parameters of the fluid were dimensionless, being defined as density (*ρ* = 1), specific heat (*c*_*p*_ = 0.71), thermal conductivity (*k* = 1), viscosity (*μ* = 0.1), and coefficient of thermal expansion (*β* = 1.408). In this problem, there is no heat generation, and the acceleration of gravity was found equal to 10 (dimensionless).

The following boundary conditions were considered: superior and inferior faces, wall conditions (*u* = 0; *v* = 0 and k∇T = h (T−T_∞_)); on the left face (*u* = 1500 and 100 and *v* = 0); on the right face, output condition ($\frac{\partial u}{\partial x}$ = 0; $\frac{\partial T}{\partial x}$ = 0; *P* = 0) and *q*’’ = 0. The initial conditions were *T* = 0, *u* = 0, *v* = 0, and *P* = 0. We worked with Reynolds dimensionless numbers equal to 1500 and 100, heat transfer coefficient equal to 0.1 and 0.01, reaching a drying air temperature of 60°C and 40°C, respectively.

The drying curves were fitted to the experimental data using thirteen different semi-empirical and empirical equations, discriminated below:

**Table:** 

Models	Models references	
RX=exp(−kt)	Newton	(5)
$RX=\text{exp}\left(-kt^{n}\right)$	Page	(6)
$RX=\text{exp}\left(-\left(-kt^{n}\right)\right)$	Page Modified	(7)
RX=aexp(−kt)	Henderson & Pabis	(8)
RX=aexp(−kt)+c	Logarithmic	(9)
$RX=a\text{exp}\;\left(-k_{o}t\right)+b\;\text{exp}\;\left(-k_{l}t\right)$	Two Terms	(10)
RX=aexp(−kτ)+(1−a)exp(−kat)	Two Exponential Terms	(11)
$RX=1+at+bt^{2}$	Wang & Singh	(12)
$RX=a\;\text{exp}\;\left(-kt\right)+b\;\text{exp}\left(-k_{0}t\right)+c\;\text{exp}\;\left(-k_{1}t\right)$	Henderson & Pabis Modified	(13)
$RX=a\;\text{exp}\;\left(-kt^{n}\right)+bt$	Midilli	(14)
RX=aexp(−kt)+(1−a)exp(−kbt)	Diffusion approximation	(15)

where

*RX*: moisture ratio (dimensionless)
*t*: drying time (h)
*k*, *k*_*o*_, *k*_*1*_: drying constant (h^-1^)
*a*, *b*, *c*, *n*: model coefficients

For determining the ratios of moisture during drying under different conditions, Eq (16) was used:

$$RX=\frac{\bar{X}-X_{e}}{X_{0}-X_{e}}$$ (16)

where

$\bar{X}$: moisture content of the product (d.b.)

*X*_*0*_: initial moisture content of the product (d.b.)

*X*_*e*_: equilibrium moisture content of the product (d.b.)

Theoretical methods were used to assess the movement of water in soybeans, which consider the external conditions and the internal mechanisms of energy and mass transfer and their effects [28–32]. According to the drying of soybeans, moisture was transported by the mechanisms of liquid diffusion, capillary diffusion, surface diffusion, hydrodynamic flow, vapor diffusion, or thermal diffusion [33–36].

In this work, we adopted the liquid diffusion mechanism as a reference. This theory has been widely used in the field of food and drying of plant products, although there are some assumptions that must be considered for its implementation, such as the reduction in the volume of discarded material, lack of capillary effect, immediate entry of the bodies in thermal equilibrium with the air, and the effects of mass and energy transference from one body to another is considered negligible. However, due to practical limitations, when liquid diffusion is used for organic products, these assumptions are generally considered satisfactory [37–40].

In the liquid diffusion theory, the moisture rate can be expressed by Fick’s second law, Eq (17):

$$\frac{\partial X}{\partial t}=3D\left(T\right)\frac{\partial^{2}X}{\partial r^{2}}$$ (17)

where

*X*: moisture content (kg_water_/kg_DS_)
*t*: time (s)
*D*: diffusivity (m^2^ s^-1^)
*r*: radius coordinate (m)

The boundary condition on the surface of the soybean grain is given by Eq (18):

$$X_{N}=X_{eq}+\left(X_{i}-X_{eq}\right)e^{-\beta t}$$ (18)

where

*ß*: drying constant at the grain surface (s^-1^)
*N*: number of radical divisions or number of experimental points (dimensionless)
*eq*: value at equilibrium

A sphere with initial moisture content that is subjected to a drying process in the open air under constant conditions can be described by Fick’s theory, Eq (19) [41, 42]:

$$RX=\frac{\bar{X}-X_{e}}{X_{0}-X_{e}}=\frac{6}{\pi^{2}}\sum_{n=1}^{\infty }\frac{1}{n^{2}}\exp\;\left(\frac{-D_{AB}n^{2}\pi^{2}t}{R^{2}}\right)$$ (19)

It is usual to consider the value of the diffusion coefficient constant or linearly dependent on the temperature of the drying air. This relationship has been expressed by the Arrhenius model, Eq (20) [43]:

$$D=A\;\exp\;\left(-\frac{E}{RT}\right)$$ (20)

where

*A*: constant (m^2^ s^-1^)
*E*: activation energy (kJ kmol^-1^)
*R*: universal gas constant (8.314 kJ kmol^-1^ K^-1^)
*T*: absolute temperature (K)

### 2.3 Experimental design

In the quality evaluation of the pulped and natural coffees after drying with heated air between 40°C and 60°C, a completely randomized experimental design with factorial (2 × 2) was employed. The drying experiments were replicated two times. The treatments consisted of two types of coffee processing (pulped grains and natural fruits) and two velocities, heat transfer coefficients, and drying air temperatures (*u* = 100 / *h* = 0.01 / 40°C and *u* = 1500 / *h* = 0.1 / 60°C). The data were analyzed using Sisvar 4.0, and the averages were compared by the Tukey test.

### 2.4 Physical–chemical and sensorial analysis

The samples to determine the moisture content of coffee in mechanical drying were collected hourly. The moisture content was determined by the oven-drying method at 105 ^±^ 3C during 24 h [44]. The electrical conductivity and leaching of potassium ions of the raw grains were determined by adapting the methodology recommended by Kryzyanowski et al. [45].

The reading was performed on a Digimed NK-2002 flame photometer. With the obtained data, the leachate potassium was calculated and the result was expressed in ppm. The titratable acidity was determined by titration with 0.1 N NaOH by methodology described AOAC [46]. The total and reducing sugars were extracted using the Lane Enyon method described by AOAC [46].

The non-reducing sugars were found by the difference between totals and reducers. The values were expressed as a percentage. The total soluble solids were determined in an ABBE bench-top refractometer, model 2 WAJ, according to AOAC [46]. The grease acidity was determined by titration, according to method 0202 A, or the rapid method of grease acidity, as described by AOAC [46].

Sensory analysis was performed by three tasters working at the Q-grader group, in the city of Alfenas–MG, Brazil. Each coffee sample consisted of five cups and one determination per sample was performed by taster.

Eight characteristics related to organoleptic standards of the beverage were evaluated according to the methodology of the Brazil Specialty Coffee Association (BSCA, 2015), overall perception, clean cup, balance, aftertaste, sweetness, acidity, body and flavor. All procedures for the preparation of the coffee beverage and sensory analysis followed the national and international rules of the Cup of Excellence (CoE) methodology adapted by BSCA [47], as well as the coffee sensory evaluation form.

In this methodology, the beverage was evaluated and the samples started with score 36. From this point, it sums up the scores of each attribute, from 0 to 8, to make up the final score. Beverage evaluation followed a grading scale from 36 to 100, in which 100 points was the maximum score. If the coffee sample reached a final score greater or equal to 80 points (80%) was considered as specialty coffee, according to BSCA [47].

### 2.5 Statistical analysis

To adjust the mathematical models of the analysis of drying grains, nonlinear regression was performed through the quasi-Newton method using the computer program Statistica 7.0^®^. To verify the degree of fit of each model, the significance of the regression coefficient was considered using theby t-test with 1% and 5% level of probability, the magnitude of the coefficient of determination (R^2^), the mean relative error values (P), and the average estimated error (SE), and the behavior of the distribution of residuals was verified (S1 Table). The relative average error and the average error estimated for each model were respectively calculated according to the following Eqs (21) and (22):

$$P=\frac{100}{n}\sum \frac{\left|Y-\hat{Y}\right|}{Y}$$ (21)

SE=∑(Y-Y^)2GLR (22)

where

*Y*: experimentally observed value
$\hat{\text{Y}}$: value calculated by the model
*n*: number of experimental observations
*GLR*: degrees of freedom of the model

The analyses of data for the physical and physical–chemical quality were performed analyzed with an analysis of variance, Tukey’s test at 1 and 5% probabilities, and linear regression. Three repetitions were performed, for each sample, and for each quality assessment.

## 3. Results and discussion

**Fig 5:**
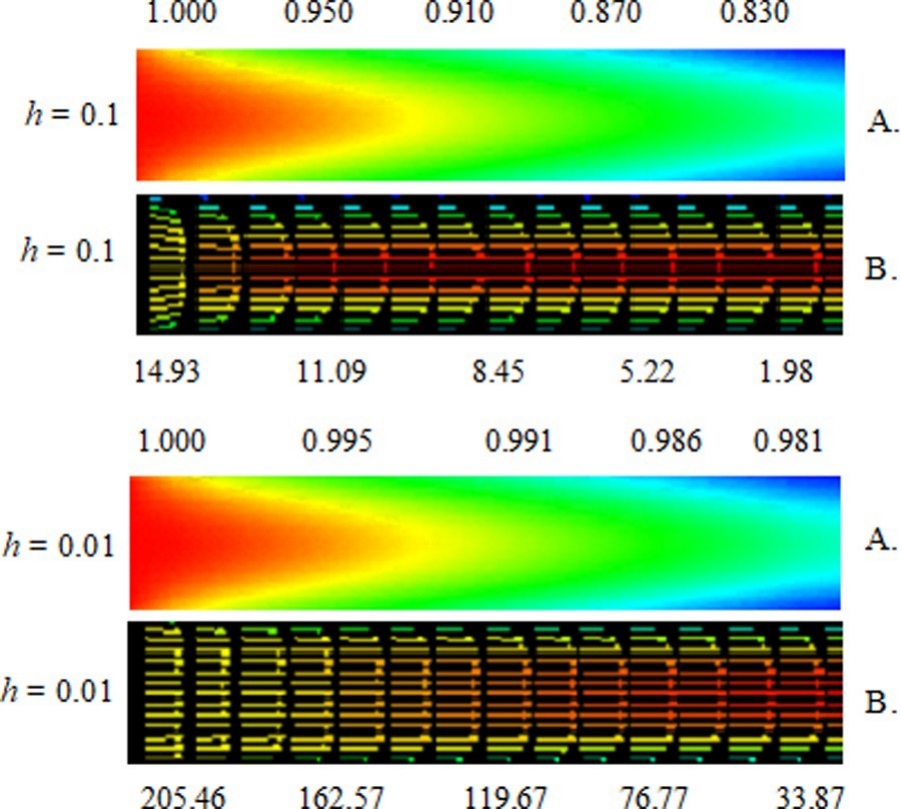
Air temperature (dimensionless) (A), air speed distribution (m s^-1^) (B), in the gas heat generator as a function of Reynolds number (speed), convective heat transfer coefficient, and drying air temperature (*u* = 1500 / *h* = 0.1 / 60°C and *u* = 100 / *h* = 0.01 / 40°C).

Fig 5A and 5B represent the distribution of temperature and air velocity controlled in the combustion chamber of the gas heat generator.

Comparing the final temperatures values in function of the heat transfer coefficient (Fig 4), it appears that the greatest temperature values were obtained when working with *h* = 0.1. This occurred because the energy losses in the upper and lower walls of the heat generator chamber grew with the decrease in the heat transfer coefficient and the Reynolds number [37, 38].

It is observed that the velocity presented a fully developed profile, in which the higher speed value lies in the central region of the outlet (Fig 4). This happened because the friction of the air in the walls of the duct reduces the speed in this region and by the law of conservation of mass, this amount of movement is transferred to the center of the duct [48, 49]. It is also observed that a higher speed value was obtained when working with higher values of the Reynolds number. Another observation is that the higher the Reynolds number and speed input are, the lower is the boundary layer. The drying air diffusion system reduced temperature variations, allowing for faster and more efficient drying.

According to the results of the air heating fluid dynamics, a PLC (analog control) with four analog inputs type 4 to 20 mA was adopted. One of them was destined for information about temperature and drying air humidity at the inlet of the dryer, and other input for information about temperature and relative humidity of the drying air at the dryer outlet. The third controller input was intended to obtain information of the mass grain temperature and the last input of the PLC to acquire information of the dynamic pressure exerted by the fan [50].

The PLC had two outlets type 4 to 20 mA, where one of them was targeted to a servomotor connected to the burner, and the other to control the gas flow. The other outlet was intended for the frequency inverter, which in turn controls the engine speed and airflow in the fan. By controlling these two variables (temperature and dynamic pressure) there was an increase in the drying process efficiency, thus reducing energy costs and increasing the quality of the end product.

The PLC controller was programmed for a lower dynamic air pressure (80 m³ min^-1^) or a lower air inlet velocity at the dryer (28 m s^-1^), i.e., if the dynamic air pressure was lower than 80 m³ min^-1^, then a message would be provided to the frequency inverter to increase the airflow into the fan. However, if the dynamic air pressure was greater than 80 m³ min^-1^, then the temperature was changed. If the temperature of the grain mass was less than 45°C and the equilibrium moisture of the grains greater than 11%, then the temperature would be increased. If the temperature of the grain mass was greater than 45°C and the equilibrium moisture content less than 11%, then the temperature would be reduced.

**Fig 6:**
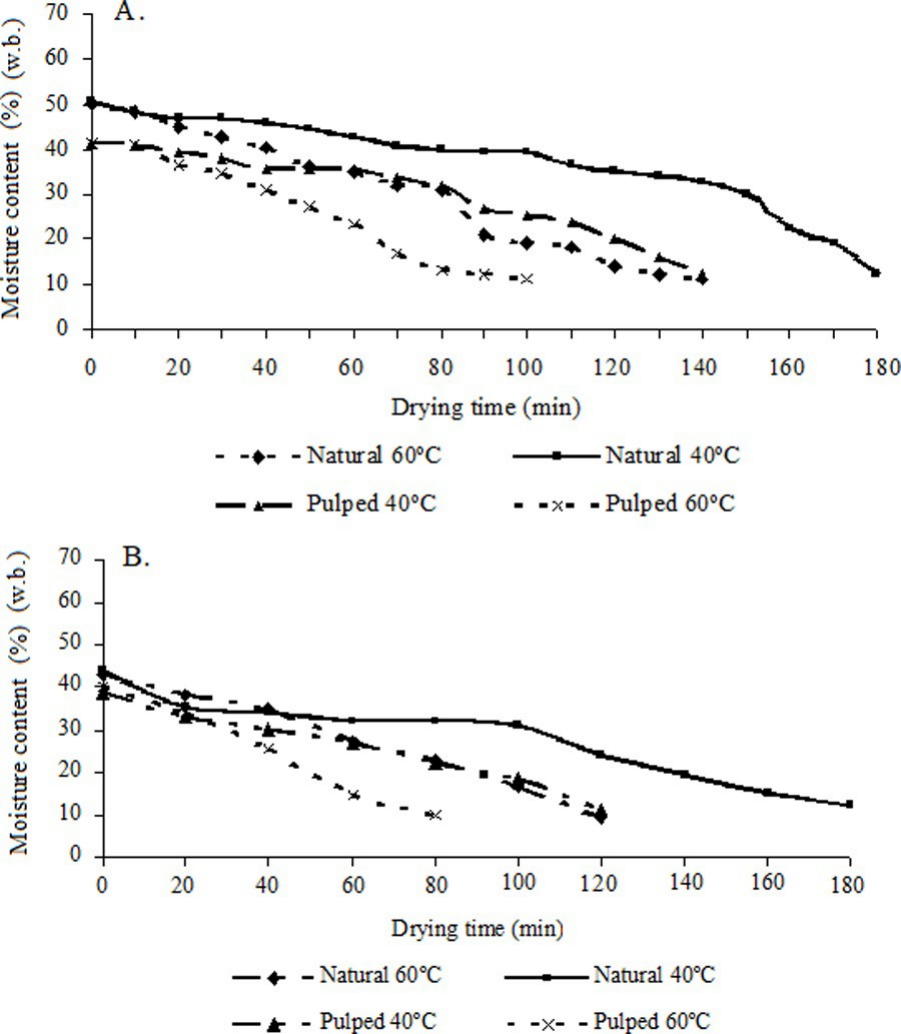
Drying curves at temperatures of 40°C and 60°C for natural and pulped coffees. First drying (A), second drying (B).

As noted in Fig 6A and 6B, the drying time was influenced by the temperature of the drying air in such a way that the higher the drying temperature was, the lower the time to complete drying, regardless of the type of processing.

When evaluating the drying of coffee in the controlled system, for both natural and pulped coffees, it was observed that the average time for drying the pulped coffee at 60°C was 1.5 h, whereas drying the natural coffee required 2.1 h. The average time for drying the pulped coffee at 40°C was 2.1 h against 3.0 h for the natural coffee. At the beginning of drying, the evaporation of the moisture content of the coffee prevents the rapid increase in temperature of the coffee mass, keeping it lower than that of the hot air. Due to the drying time, it was observed that the increase in the drying speed and uniformity of the process reduced the energy consumption three times in relation to the conventional system.

When the coffee is at a more advanced stage of drying, the air and coffee temperatures approach owing to the difficulty of moisture migration from the internal parts to the outside of the fruits. The heated air makes it less efficient the entraining of moisture from the grains owing to the greater tension with which water is removed. Thereafter, to obtain a good quality coffee and a soft beverage product, it is necessary to maintain the drying temperature around 45°C in the coffee mass [1–3, 51, 52].

The mean values of temperature and relative air humidity in the inlet of the dryer with air heated between 40°C and 60°C for each treatment and the two replicates are listed in Table 1. As can be found in this table, the data of average relative humidity and air temperature were similar for all the tests, ranging from 49% to 61% (w.b.) and 20.23°C to 23.43°C, respectively.

**Table 1:** Mean values of the product and ambient air conditions for all treatments.

R	Coffee	T (°C)	Air flow(m³ min^-1^ m²)	Moisture content (%) (w.b.)		Air conditions at the dryer inlet	
				Start	End	T (°C)	UR (%)
I	Pulped	60	20	41.34	11.35	20.76	61
	Natural	60	20	50.11	11.15	20.76	61
	Pulped	40	20	41.34	12.00	20.23	59
	Natural	40	20	50.95	12.22	20.23	59
II	Pulped	60	20	40.12	9.33	21.54	55
	Natural	60	20	43.08	9.49	21.54	55
	Pulped	40	20	38.71	11.12	21.89	54
	Natural	40	20	43.87	12.22	21.89	54

It was also found that the initial moisture content of the pulped coffee ranged from 50.78% to 57.66% (w.b.) and, after drying, the moisture content was 10.15% (w.b.). For the natural coffee, it was observed that at the beginning of the drying, the moisture content ranged from 58.60% to 65.60% (w.b.), reaching the average moisture of storage at 12% of moisture content (w.b.).

Before passing through a drying process with heated air at 40°C and 60°C, in the yard, both coffees were submitted to a pre-drying for two days, so that the two types could start the mechanical drying at the same environmental conditions, losing, on average, five percentage points of moisture content. It was observed that the natural coffee began the drying with a moisture content ranging from 43.08% to 50.95% (w.b.), reaching an average storage moisture content at 11.03% (w.b.), whereas the pulped coffee started with moisture contents ranging from 38.71% to 40.42% (w.b.), reaching the average moisture of storage at 10.56% (w.b.).

Table 2 presents the coefficients of the models adjusted for the natural and pulped coffees analyzed during drying at different drying air temperatures.

**Table 2:** Parameters obtained from models fitted to the data for drying of natural and pulped coffees processed with different temperatures of drying air.

Mathematical models								
Equations	Process	T (°C)	*k*					
Newton	Pulped	40	0.11165					
		60	0.36466					
	Natural	40	0.09313					
		60	0.33476					
		T (°C)	*k*	*n*				
Page	Pulped	40	0.06506	1.24176				
		60	0.37145	0.98406				
	Natural	40	0.02064	1.64519				
		60	0.36838	1.00382				
		T (°C)	*k*	*n*				
Page Modified	Pulped	40	0.11070	1.18175				
		60	0.36554	0.98406				
	Natural	40	0.09456	1.64525				
		60	0.33624	0.97149				
		T (°C)	*a*	*k*				
Henderson and Pabis	Pulped	40	1.03076	0.115350				
		60	1.00641	0.367040				
	Natural	40	1.0904	0.10252				
		60	0.9965	0.33358				
		T (°C)	*a*	*k*	*c*			
Logarithmic	Pulped	40	1.23956	0.07663	-0.23818			
		60	0.98021	0.40651	0.03651			
	Natural	40	3.00380	0.02194	-1.97945			
		60	0.98510	0.34738	0.01543			
		T (°C)	*a*	*K*_*0*_	*b*	*k*_*1*_		
Two terms	Pulped	40	0.51537	0.11535	0.51537	0.11534		
		60	0.50321	0.36704	0.50321	0.36704		
	Natural	40	0.10752	0.10252	0.54520	0.10252		
		60	0.49829	0.33358	0.49829	0.33358		
		T (°C)	*a*	*k*				
Two exponential terms	Pulped	40	1.77245	0.15702				
		60	0.55580	0.48030				
	Natural	40	0.00707	12.8223				
		60	0.61493	0.40721				
		T (°C)	*a*	*b*				
Wang and Sing	Pulped	40	-0.08810	0.00209				
		60	-0.28150	0.02143				
	Natural	40	-0.06070	0.00039				
		60	-0.26310	0.01890				
		T (°C)	*a*	*k*	*b*	*k*_*0*_	*c*	*k*_*1*_
Henderson and Modified Pabis	Pulped	40	0.34358	0.11534	0.34358	0.11534	0.34358	0.11534
		60	0.33547	0.36704	0.33547	0.36704	0.33547	0.36704
	Natural	40	0.36346	0.10252	0.36346	0.10252	0.36346	0.10226
		60	0.33219	0.35358	0.33219	0.33358	0.33219	0.33358
		T (°C)	*a*	*k*	*n*	*b*		
Midilli	Pulped	40	0.97144	0.05386	1.31332	0.00018		
		60	1.00690	0.36186	1.10794	0.00751		
	Natural	40	0.98716	0.02184	1.52303	-0.00597		
		60	1.00236	0.34825	0.97495	0.00042		
		T (°C)	*a*	*k*	*b*			
Diffusion approximation	Pulped	40	-4.03326	0.21346	0.86256			
		60	0.08348	0.11058	3.69000			
	Natural	40	-6.59768	0.22287	0.86600			
		60	0.13864	0.62679	0.49026			

Among the models that provided good results, the Midilli model was selected to represent the phenomenon of drying coffee owing to its number of significant coefficients that described the results consistently. It was observed that the magnitude of the drying constant (k) for the Midilli model, which represents the effect of external conditions on drying, increases linearly with the rise in temperature of the drying air (Table 2).

The coefficient of determination was above 99% (Table 3), which according to Madamba et al. [53] indicates a satisfactory representation of the phenomenon under study. According to this researcher, the use of the coefficient of determination as the only evaluation criterion for the selection of nonlinear models is not a good parameter to represent the drying phenomena.

**Table 3:** Coefficients of determination (R^2^), mean relative errors (P), mean estimated errors (SE), and distribution of residuals for the models analyzed during drying of natural and pulped coffee under different temperatures.

Mathematical models	Pulped coffee		Natural coffee	
	40°C	60°C	40°C	60°C
R^2^ (%)				
Newton	51.31	93.86	96.45	99.81
Page	65.68	96.20	99.51	99.82
Page Modified	52.10	62.00	99.51	99.82
Henderson and Pabis	58.23	94.25	97.19	99.81
Logarithmic	58.35	67.15	99.47	99.82
Two Terms	65.27	94.25	97.19	99.81
Two exponential terms	70.85	93.77	96.31	99.82
Wang and Singh	57.38	98.12	99.44	99.31
Henderson and Pabis Modified	69.03	94.25	97.19	99.81
Midilli	99.37	99.84	99.65	99.82
Diffusion approximation	67.08	96.36	99.23	99.82
P (%)				
Newton	25.40	7.13	11.74	19.57
Page	0.09	0.04	11.62	19.36
Page Modified	74.32	10.47	11.62	19.37
Henderson and Pabis	30.79	1.56	0.88	1.47
Logarithmic	14.63	90.02	0.54	0.89
Two Terms	3.51	1.62	0.88	1.47
Two exponential terms	5.41	3.20	2.97	4.96
Wang and Singh	17.35	7.51	8.26	13.77
Henderson and Pabis Modified	53.79	1.25	0.88	1.47
Midilli	0.01	0.03	0.55	0.24
Diffusion approximation	4.75	9.42	12.60	21.00
SE (decimal)				
Newton	0.1357	0.0773	0.0880	0.0682
Page	0.1245	0.0002	0.0347	0.0553
Page Modified	0.2618	0.1837	0.0347	0.0553
Henderson and Pabis	0.5359	0.0767	0.0767	0.0721
Logarithmic	0.1297	0.1407	0.0352	0.0575
Two Terms	0.1248	0.0848	0.0886	0.1020
Two exponential terms	0.1287	0.0813	0.0951	0.0544
Wang and Singh	0.1352	0.0451	0.0374	0.0720
Henderson and Pabis Modified	0.1195	0.0962	0.1085	0.1442
Midilli	0.0137	0.0535	0.0335	0.0727
Diffusion approximation	0.1350	0.0656	0.0467	0.0618
Distribution of residue				
Newton	A	A	A	A
Page	A	A	A	A
Page Modified	T	A	A	A
Henderson and Pabis	T	A	A	A
Logarithmic	A	T	A	A
Two Terms	A	A	A	A
Two exponential terms	A	A	A	A
Wang and Singh	A	A	A	A
Henderson and Pabis Modified	A	A	A	A
Midilli	A	A	A	A
Diffusion approximation	A	A	A	A

**Fig 7:**
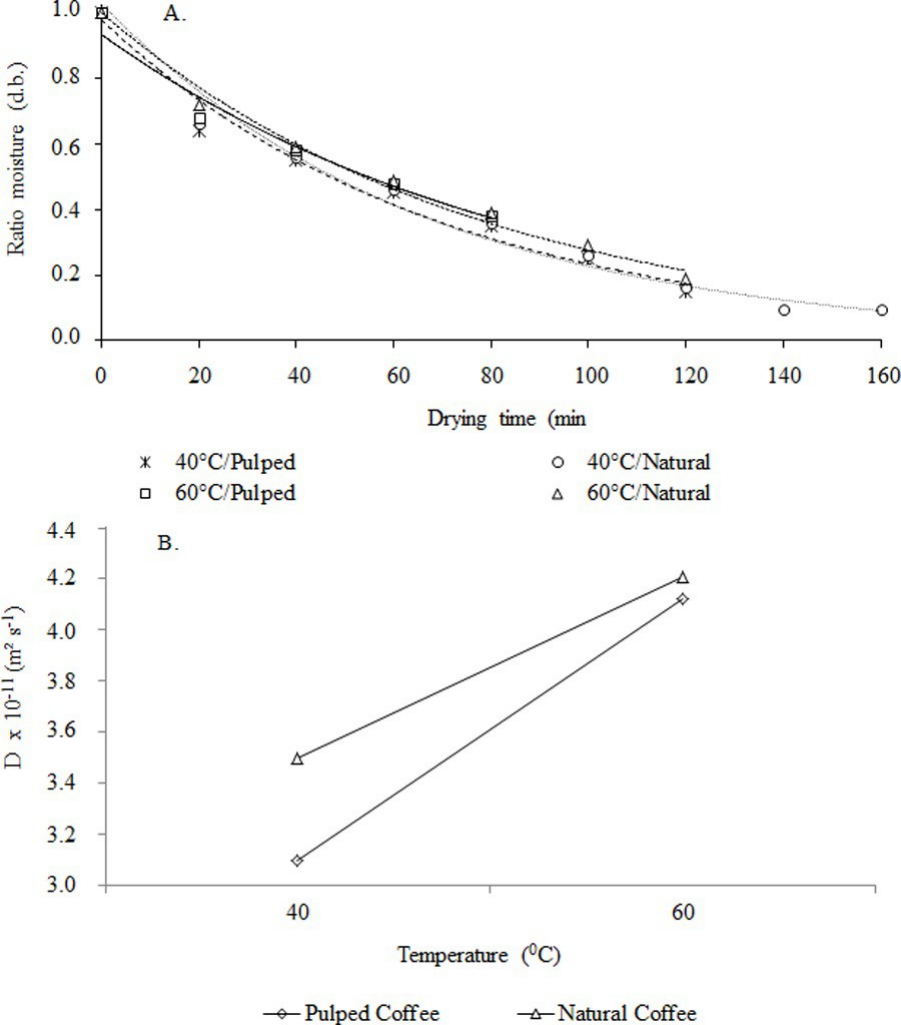
Moisture ratios adjusted by the Midilli model (A), values for the effective diffusion coefficient (B) due to different air temperatures in the drying of natural and pulped coffee.

The estimated average error to Wang & Singh, Page, logarithmic approximation of diffusion, Midilli, and exponential for two terms models showed lower values for drying in different temperatures. However, the models Page, diffusion approximation, and Midilli showed a low average error estimated [54]. The Midilli model was better that the other evaluated because it presented higher R² for all. The correlations between the experimental and predicted data of the Midilli model for drying natural and pulped coffee are shown in (Fig 7A). The moisture ratio decreased with time and the difference between the moisture ratios increased continuously from the beginning to the end of drying. The observed and predicted values of the model are perfectly consistent and are almost the same [30, 33].

Fig 7B appears that during the drying the effective diffusion coefficient increased significantly (P<0.05), with the increase of the temperature (Fig 2A). The linear fit obtained indicates a uniform variation of diffusivity with temperature, the value being the variation of diffusivity coefficient obtained at 60°C, slightly higher than the temperature 40°C (Fig 2A). A variation in the effective diffusion coefficient occurred with increasing temperature, which increased the molecular vibration of water molecules and contributes to a faster diffusion. These values are within the range of diffusivities (10^−11^ to 10^−9^ m^2^ s^-1^) for different crops using several drying methods [55, 56]. In convective drying, a high drying air temperature transfers heat to the product, resulting in a higher product temperature, and then, the water within the product moves by diffusion and evaporates into the drying air [5, 14, 57]. The mass transfer process will stop when the water vapor pressure at the product surface becomes equal to the water vapor pressure of the drying air, thus entering hygroscopic balance. Therefore, the drying air temperature has an essential effect on the drying of pulped coffee.

The uniformity and distribution of heat and drying air increased the quality of the final coffee. In this way, the effects of the types of drying and processing on the coffee quality were observed (Table 4). The drying temperature of 60°C compromised the structures of the cell membranes, which led to easy deterioration of the coffee. As the drying air temperature increased, there was an increase in leached ions, electrical conductivity, titratable acidity, grease acidity, and soluble solids. Among the types of processing, higher values of leached ions, electrical conductivity, titratable acidity, grease acidity, and soluble solids were observed for natural coffee cherries [13, 18, 51, 58]. The results obtained prove the reduction of the quality of the coffees with the increase of the drying air temperature (Table 4).

**Table 4:** Quality of naturally processed and pulped coffees after drying at different temperatures.

Drying air temperature (°C)	Processing type		Processing type	
	Natural	Pulped	Natural	Pulped
	Electrical conductivity (μS cm^-1^ g^-1^)		Potassium leaching (ppm)	
40	130.00 Ba	93.33 Bb	40.00 Ba	32.00 Bb
60	230.00 Aa	215.80 Ab	66.33 Aa	68.33 Aa
	Reducing sugars (%)		Total sugars (%)	
40	0.64 Aa	0.35 Ab	8.33 Aa	8.33 Aa
60	0.57 Ba	0.27 Bb	8.00 Ba	7.33 Ba
	Total titratable acidity (NaOH 0.1N/100g)		Fatty acidity (KOH 0.1N/100g)	
40	171.33 Ba	179.67 Ba	1.22 Ba	1.08 Ba
60	216.67 Aa	208.33 Ab	2.61 Aa	1.54 Ab
	Soluble solids (%)		Sensory analysis (scores, %)	
40	33.00 Aa	27.00 Ab	80.22 Ab	84.33 Aa
60	33.33 Aa	26.00 Ab	68.11 Bb	74.67 Ba

Means followed by the same lowercase letter in the row and uppercase letters in the column did not differ at 1 and 5% probability.

The sugary mucilage that surrounds the natural coffee cherry is an ideal substrate for the development of fungi and bacteria, which can alter the physical–chemical composition and consequently the final quality of the beverage. For this reason, another positive factor in the wet process is the obtention of better quality coffees, which maintain their body, sweetness, and aroma characteristics.

In a study on the quality of coffee prepared under different processing methods, superior characteristics of the beverage were observed for peeled, pulped, and demucilated coffees in relation to natural coffee [1, 54, 58]. In comparison with pulped coffee, natural coffee needed a longer drying time to reach the desired moisture content for storage (Fig 6). The effects of drying time on the quality of the coffees were most evident at the drying air temperature of 60°C (Table 4).

A significant increase (P<0.01) of electrical conductivity and potassium leaching were observed as a function of the increase in the drying temperature. The quantities of ions leached were mainly due to the high temperature of drying, which interferes with the integrity of cell membranes. The same occurs with the electrical conductivity. The values obtained in the sensory analysis of natural and pulped coffees regarding the types of drying are presented in Table 4.

It was verified that the increase in the drying temperature of natural coffee resulted in the smallest notes of the sensory analysis. The quality of the coffee is involved with related to the flavor and aroma of the beverage, and this is due to the complexity of the coffee compounds. In the drying of natural coffee cherries, the best grades were obtained in the yard, while the worst ones were attained for coffee dried at 60°C [13, 51]. Some authors evaluated the influence of mechanical drying at 40°C, in comparation sun drying on the quality of Arabica coffee and verified that the drying process affected levels of some amino acids. Sucrose content was higher in mechanical drying at 40°C. Combined results of sensory and chemical analyses showed that the drying at 40°C was superior for preserving overall flavor quality [13, 58].

The drying air temperature is the most flexible parameter in a high temperature drying system, significantly influencing the drying rate and efficiency, as well as the final quality of the product. If not controlled, the drying air temperature causes physical damage, such as discoloration of grains, breaks, and cracks [2–7].

These effects are caused, among other reasons, by the lower values of electrical conductivity, potassium leaching, titratable acidity, grease acidity, and the higher values of reducing and total sugars [4, 6, 7]. The electrical conductivity and potassium leaching are indicators of the integrity of cell membranes, and the sugars are involved in the protection mechanisms of membranes. It can be considered that drying on yard can contribute to the development of protective mechanisms for the cell membranes, preserving their integrity, and hence, maintaining the quality of the coffee. Some authors verified in studies that the cell membranes of coffee beans are damaged when the moisture contents of coffee are between 25% to 20% (w.b.), using drying temperature above 50°C for pulped and natural coffee [2–5, 58].

The decrease in coffee quality is also associated with increased acidity, which is mainly due to the number of defects in the beans caused by drying. The increase in acidity has also been attributed to fermentation during the drying process and the concentration of acids resulting from the degradations caused by the drying temperature [5, 13, 18]. With peeled and pulped grains, the variation in temperature and relative humidity of the drying air had less influence on the titratable acidity, while the cherry fruits showed a reduction in the acidity indexes with the elevation of the drying air temperature [1–3].

The pulped coffee, compared to the natural one, presented a better taste and aroma. Studies about the quality of coffee prepared under different processing methods reported superior characteristics for peeled, pulped, and without mucilage coffees. The beverage quality is also related to fatty acids. The grease acidity decreases with lower drying air temperature, regardless of the type of processing, affecting less the beverage quality [3, 6, 54].

Studies have evaluated the quality of coffee for export produced at different altitudes associated with the effects of processing and drying. Production altitudes above 1000 m have obtained the best sensory results, in terms of aroma and flavor, with superior qualitative attributes, when associated with drying with temperatures below 40°C [59, 60]. High drying temperatures and high moisture reduction rates degrade the structure of the coffee and the cell membranes, causing extravasation and oxidation in the oils, and thus increasing the levels of fatty acids. The composition of fatty acids depends on some factors such as the coffee species and varieties, which may undergo hydrolysis reactions of triacylglycerols. The release of fatty acids forms “off-flavor” oxidations in the chemical makeup of coffee during drying [2, 3, 57, 61, 62].

Sugars are related to the beverage quality and the quantities of these components mainly depend on the species and place of cultivation of the coffee, in addition to the stage of maturation of the fruits [57, 61]. However, post-harvest operations can cause variations in sugar levels. The natural coffee cherry has higher levels of reducing sugars, compared to the pulped coffee [1, 2, 54, 58]. This can be explained by the presence of the husk and mucilage in the coffee during drying, which are rich in sugars, and translocations of these chemical components into the grain can occur.

The variations and the increase in drying temperature is detrimental to the sensory attributes of the coffees. Some authors studied the effects of bean mass temperature on sensory quality, reported that the increase in drying temperature was detrimental to the maintenance of sensory quality of parchment and natural coffee [1, 57, 61].

## 4. Conclusions

A reduction in the heat transfer coefficient resulted in an increase in temperature and a higher air speed in the heat generator. The adjustments of fluid dynamics in the burning of gas and the application of a heated air diffuser had a significant influence on the reduction of drying time, allowing adjustments in the moisture ratio curves and the effective diffusion coefficient with the increase in drying air temperature and better results in the final quality of naturally processed and pulped coffees.

The increase in drying air temperature decreased the drying time, increased the diffusivity, and reduced the final quality of the coffees. Better quality after drying was obtained with pulped coffee. Thus, it is concluded that the adapted technological set, a rotary dryer with gas heating and diffusion of the heated air, had a high performance regarding the final quality of the coffees, and for this reason it is recommended to producers and the industry. The drying system optimization allowed continuity and uniformity of the process, reduced the time and increased the final quality of the coffees.

## Supporting information

S1 Table Data set as a supporting information. (XLS)

S1 Graphical abstract (TIF)
