# Supplementary figures and images for: Development and validation of a heated drying air diffusion system to optimize rotary dryers and final coffee quality
Source: PLoS One. 2021 Jun 22;16(6):e0251312. doi: 10.1371/journal.pone.0251312 (PMC8219142; doi:10.1371/journal.pone.0251312)

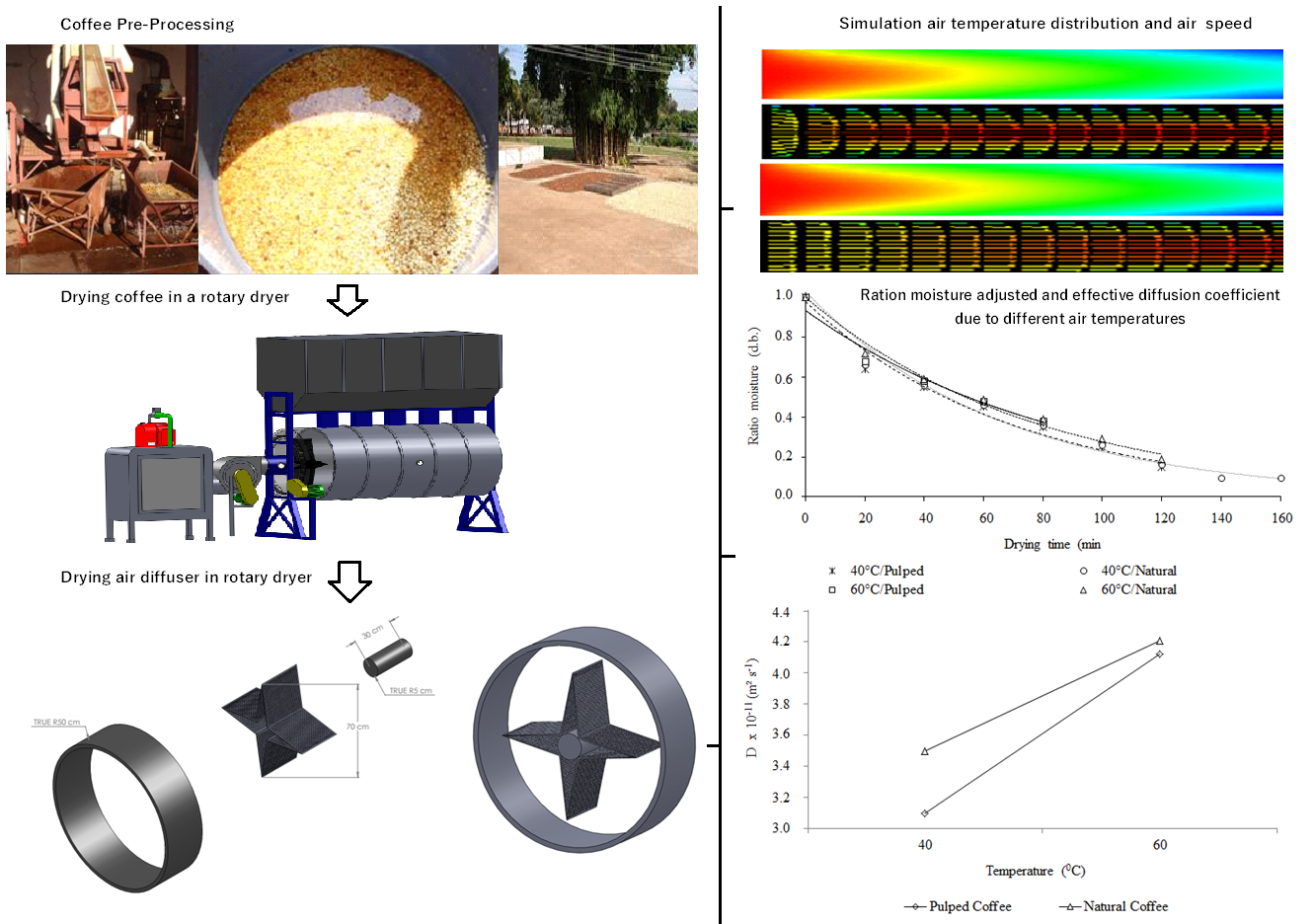

Supplement: S1 Graphical abstract — (TIF) [file pone.0251312.s002.tif]
